# Accuracy of Elastic Fusion of Prostate Magnetic Resonance and Transrectal Ultrasound Images under Routine Conditions: A Prospective Multi-Operator Study

**DOI:** 10.1371/journal.pone.0169120

**Published:** 2016-12-29

**Authors:** Paul Moldovan, Corina Udrescu, Emmanuel Ravier, Rémi Souchon, Muriel Rabilloud, Flavie Bratan, Thomas Sanzalone, Fanny Cros, Sébastien Crouzet, Albert Gelet, Olivier Chapet, Olivier Rouvière

**Affiliations:** 1 Hospices Civils de Lyon, Department of Urinary and Vascular Radiology, Hôpital Edouard Herriot, Lyon, France; 2 Hospices Civils de Lyon, Department of Radiation Oncology, Centre Hospitalier Lyon-Sud, Pierre-Bénite, France; 3 Hospices Civils de Lyon, Department of Urology, Hôpital Edouard Herriot, Lyon, France; 4 Inserm, U1032, LabTau, Lyon, France; 5 Hospices Civils de Lyon, Service de Biostatistique et Bioinformatique, Lyon, France; 6 Université de Lyon, Lyon, France; 7 CNRS, UMR5558, Laboratoire de Biométrie et Biologie Evolutive, Equipe Biostatistique-Santé, Villeurbanne, France; Chinese Academy of Sciences, CHINA

## Abstract

**Purpose:**

To evaluate in unselected patients imaged under routine conditions the co-registration accuracy of elastic fusion between magnetic resonance (MR) and ultrasound (US) images obtained by the Koelis Urostation^™^.

**Materials and Methods:**

We prospectively included 15 consecutive patients referred for placement of intraprostatic fiducials before radiotherapy and who gave written informed consent by signing the Institutional Review Board-approved forms. Three fiducials were placed in the prostate under US guidance in standardized positions (right apex, left mid-gland, right base) using the Koelis Urostation^™^. Patients then underwent prostate MR imaging. Four operators outlined the prostate on MR and US images and an elastic fusion was retrospectively performed. Fiducials were used to measure the overall target registration error (TRE_3D_), the error along the antero-posterior (TRE_AP_), right-left (TRE_RL_) and head-feet (TRE_HF_) directions, and within the plane orthogonal to the virtual biopsy track (TRE_2D_).

**Results:**

Median TRE_3D_ and TRE_2D_ were 3.8–5.6 mm, and 2.5–3.6 mm, respectively. TRE_3D_ was significantly influenced by the operator (p = 0.013), fiducial location (p = 0.001) and 3D axis orientation (p<0.0001). The worst results were obtained by the least experienced operator. TRE_3D_ was smaller in mid-gland and base than in apex (average difference: -1.21 mm (95% confidence interval (95%CI): -2.03; -0.4) and -1.56 mm (95%CI: -2.44; -0.69) respectively). TRE_AP_ and TRE_HF_ were larger than TRE_RL_ (average difference: +1.29 mm (95%CI: +0.87; +1.71) and +0.59 mm (95%CI: +0.1; +0.95) respectively).

**Conclusions:**

Registration error values were reasonable for clinical practice. The co-registration accuracy was significantly influenced by the operator’s experience, and significantly poorer in the antero-posterior direction and at the apex.

## Introduction

Multiparametric magnetic resonance imaging (MRI) is increasingly performed to localize aggressive cancer prior to prostate biopsy [1–4]. However, the best method for targeting biopsies remains unclear. Direct in-bore biopsy is limited by cost and availability. Cognitive guidance is inexpensive, but the extrapolation from magnetic resonance (MR) to transrectal ultrasound (TRUS) images may be problematic because MR and ultrasound (US) images are not acquired along the same plane. MR/US fusion software may overcome this difficulty by providing a co-registration of MR and US images [5, 6].

MR/US fusion is increasingly used for targeted prostate biopsy, and series of hundreds of patients have been published during the last three years, with excellent results as compared to systematic biopsy [1, 6–14]. Yet, data on the accuracy of existing co-registration techniques is surprisingly scarce. Most manufacturers claim target registration errors (TRE) of 1–3 mm, at least when using an elastic fusion [15–20]. However, these data are based on pre-clinical studies that have several limitations. Some used only prostate phantoms. Others used MR-TRUS image pairs obtained in patients the selection of whom is not specified. They are likely to have used images of good quality, thereby inducing a selection bias. Moreover, the fusion is usually performed by highly-specialized researchers in conditions that do not fully mimic daily routine. Furthermore, comparisons of MR-TRUS image pairs used anatomical landmarks that are questionable because of their size or lack of conspicuity on MR or US images.

Because of these intrinsic limitations, the fusion accuracy actually achieved in daily routine may be quite different from the 1–3 mm error reported in pre-clinical studies. The lack of reliable data on fusion accuracy in routine becomes problematic. For example, it is needed to define a rationale management of patients with negative targeted biopsy results or to assess the optimal safety margin for focal treatments that are increasingly performed under MR/US fusion [21, 22].

Recently, fiducial markers easily visible on US and MR became available. The aim of the present study was to evaluate the accuracy of elastic MR/US fusion in unselected patients imaged under routine conditions, using these fiducials as co-registration markers.

## Materials and Methods

### Study population

The study has been approved by our institutional review board (Comité de Protection des Personnes Sud-Est IV; décision L14-46). Fifteen patients who were referred for placement of fiducial markers before external beam radiotherapy for prostate cancer and who gave written consent were prospectively included between March 2014 and May 2015.

### Principles of the MR/US fusion performed in our study

The biopsy platform is composed of a Sonoace X8 US scanner fitted with a 3D endorectal probe (3D-4-9ES, Medison, Seoul, South Korea), and connected to the Urostation^™^ workstation (Koelis, La Tronche, France).

During a regular biopsy procedure, the elastic MR/US fusion is performed in several steps. First, the operator outlines the prostate on MR images and places a spherical region-of-interest (ROI; the ‘MR target’) in the center of each MR lesion to be biopsied. Then, an axial and two oblique 3D TRUS acquisitions are obtained and fused to create an extended volume of the prostate (the ‘panorama volume’). The prostate is outlined on the panorama volume and the prostate outlines obtained on MR and US images are co-registered using elastic fusion. The MR target becomes then visible within the panorama volume.

After each biopsy, and when the needle is still in the prostate, the operator obtains a 3D axial TRUS acquisition centered on the biopsy needle. A 20-mm long rectangular ROI is then used to mark the position of the biopsy needle tip within the US volume. This new US volume is automatically co-registered within the panorama volume, and the position of the rectangular ROI featuring the biopsy needle becomes visible in the panorama volume.

The Urostation Organ Based Tracking^™^ workflow used to fuse 3D US acquisitions is based on a fully automatic registration pipeline [23]. The pipeline first uses a probe kinematic model that integrates rectal and image constraints, to determine plausible solutions of the position of the probe according to the organ. Rigid transformations between a reference volume and new acquisitions are then computed with the previous solutions by using a parametric optimization method. The best result is finally selected as an estimate to compute the elastic deformations between the reference and the new acquisitions. At each step, an image distance measure is computed to determine the best results.

The fusion between MR and US acquisitions uses a non-rigid surface registration method based on surface prostate mesh built from MRI and TRUS volumes [24].

### Placement of markers and acquisition of prostate US images

All patients included in the study were imaged on the Sonoace X8 scanner of the Urostation^™^ platform, using the 3D-4-9ES endorectal probe. The prostate volume was calculated using the ellipsoid formula. Three markers, visible at MRI and US, with a 5-mm length and a 1-mm diameter (FusionCoils^™^, Cortex Manufacturing Inc., USA) were inserted in the prostate under TRUS guidance. They were positioned in three standardized locations: the paramedial part of the right base (‘base marker’) and right apex (‘apex marker’), and the lateral part of the left mid-gland (‘mid-gland marker’) [25, 26].

One axial and two oblique 3D US acquisitions were obtained and fused to obtain the panorama volume. Then, the operator performed one 3D axial US acquisition centered on each marker, as if they were targeted at biopsy. These 3D axial US acquisitions were stored in the Urostation^™^. The markers were inserted by a total of 4 radiologists from our department of uroradiology, as part of their routine work. These radiologists had 18 (OR), 7 (FB), 2 (TS) and 2 (PM) years of expertise in prostate imaging. They inserted markers in 7, 4, 1 and 3 patients respectively.

### Prostate MR imaging

**Fig 1:**
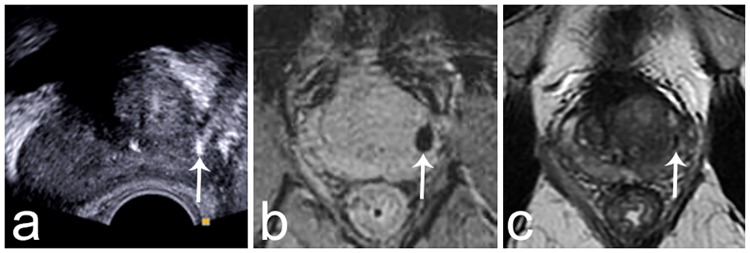
Images showing a fiducial marker implanted in the peripheral zone of the left mid-gland. A) Axial transrectal ultrasound image. The marker is markedly hyperechoic (arrow). B) Axial T2-weighted gradient echo image. The marker creates a void artifact that helps localizing it (arrow). C) Axial T2-weighted Turbo-Spin Echo image obtained at the same location as the gradient echo image. The marker is visible as a thin hyposignal (arrow).

The patients underwent 3T prostate MRI (MR750, General Electric Medical Systems, Milwaukee, WI, USA) the same day as the markers’ insertion except one who underwent MRI 5 days later (Table 1). T2-TSE axial images were then transferred to the Urostation^™^ for MR/US fusion (Fig 1).

**Table 1:** Prostate Magnetic Resonance Imaging acquisition parameters.

	Sagittal T2-TSE	Axial T2-TSE	Axial T2-GRE
Repetition time (ms)	4452	4223	640
Echo Time (ms)	145	145	20
Field of view (mm x mm)	220 x 220	220 x 220	240 x 240
Acquisition matrix	384 x 256	384 x 256	256 x 224
Flip angels (degrees)	146	146	15
Slice thickness (mm)	3	3	3
Slice spacing (mm)	0	0	0

T2-TSE: T2-weighted Turbo-Spin Echo imaging; T2-GRE: T2-weighted gradient echo imaging

### MR/US Fusion

**Fig 2:**
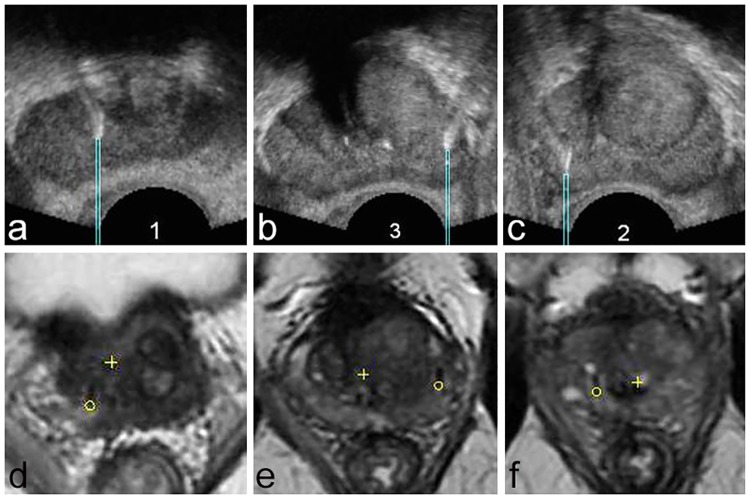
Images showing the three markers with virtual biopsies and MR targets (same patient as in Fig 1). A-C) Axial transrectal ultrasound images showing the markers implanted in the right base (A), the left mid-gland (B) and the right apex (C). A rectangular region of interest (‘virtual biopsy’) has been placed at the posterior extremity of each marker. This region of interest represents a virtual biopsy the tip of which would have reached the posterior extremity of the marker. The coordinates of the tip and base of the virtual biopsies were registered for each marker. D-F) Axial T2-weighted Turbo-Spin Echo images showing the markers implanted in the right base (D), the left mid-gland (E) and the right apex (F). A circular region of interest (‘MR target’) has been placed at the extremity of each marker. The coordinates of the center of these MR targets were registered for each marker. In case of perfect fusion, the tip of the virtual biopsies must correspond to the center of the MR targets.

Operators 1 and 2 working in consensus reviewed the 3D US acquisitions that targeted each marker. Using the Urostation^™^, they retrieved the 3D axial US acquisitions centered on each marker, and placed at the extremity of each marker the 20-mm long rectangular ROI featuring a biopsy needle the tip of which would have reached the marker’s extremity. This ROI will be referred to as ‘virtual biopsy’ (Fig 2a–2c). After automatic US/US co-registration of the 3D US acquisitions onto the panorama volume, the coordinates of the tip and base of the three ‘virtual biopsies’ in the panorama volume were calculated, using the center of the US probe as reference.

Then, the same operators reviewed the T2-TSE images and placed the center of a circular ROI on the same extremities of the three markers. These circular ROIs will be referred to as ‘MR targets’ (Fig 2d–2f).

Then, operators 1–4 independently outlined the prostate on T2-TSE images and on the US panorama volume, and an elastic fusion was obtained. The Urostation^™^ software was then used to calculate the spatial coordinates of the centers of the MR targets in the panorama volume. In case of perfect fusion, the coordinates of MR targets and virtual biopsies tips should be identical.

Operators 1 (OR) and 2 (PM) were senior uroradiologists with respectively 18 and 2 years of experience in prostate imaging. Operator 3 (CU) was a radiation physicist with 5 years of expertise in contouring prostate MR images. Operator 4 (ER) had 1 year of expertise as a senior urologist and had received a 6-month training in prostate imaging during his last year of residency.

### Target registration errors

First, we calculated the overall TRE (TRE_3D_), defined as the Euclidean distance between the center of the MR target and the tip of the corresponding virtual biopsy.

The TRE_3D_ vector was then decomposed into three vectors respectively parallel to the antero-posterior (AP) direction (direction parallel to the axis of the US probe, i.e. AP direction of the 2D US axial image), the right-left (RL) direction (direction of the 2D US axial image orthogonal to the AP axis, i.e. RL direction of the 2D US axial image), and the head-feet (HF) direction (orthogonal to the axis of the US probe and to the 2D US axial plane). The magnitudes of these vectors (TRE_AP_, TRE_RL_ and TRE_HF_) were calculated.

Finally we calculated the distance between the biopsy track axis (calculated using the coordinates of the tip and base of the virtual biopsy) and the MR target in the plane perpendicular to the biopsy track. This distance will be referred to as TRE_2D_.

### Fiducial localization error estimation

Operators 1 and 2 defined a second time the position of the virtual biopsies and MR targets, four months after their first placement. The virtual biopsies were automatically co-registered into the panorama volume and the Euclidean distance between the coordinates of their tips on the first and second placements was used to estimate the US fiducial localization error (FLE_US_). The MR targets were co-registered into the panorama volume using the outlines made by operator 1. The Euclidean distance between their centers on the first and second placements was defined as FLE_MR_.

### Statistical analysis

TRE and FLE distributions were described by the quartiles. A linear mixed model with a random intercept was used to model TRE_3D_. The mixed model allowed taking account of the correlation of the measurements carried out in a same patient and quantifying the effect of the operators, the locations of the marker and the prostate volume introduced as fixed effects. A second linear mixed model was built to model TRE_AP_, TRE_RL_ and TRE_HF_. The model allowed taking account of two random effects (patient and operator) and quantifying the effect of the direction of the vector, after adjustment on prostate volume and on location of the marker. The analysis was carried out using the package lme4 version 1.1–10 of the R software, version 3.1.3 (The R Foundation for Statistical Computing). Statistical significance was retained for a p-value < 0.05.

## Results

The patients’ median age and prostate volume were 69 years (interquartile range (IQR), 66.5–75.5) and 35 cc (IQR, 30–41) respectively. Two patients had history of transurethral resection of the prostate (S1 Table).

**Fig 3:**
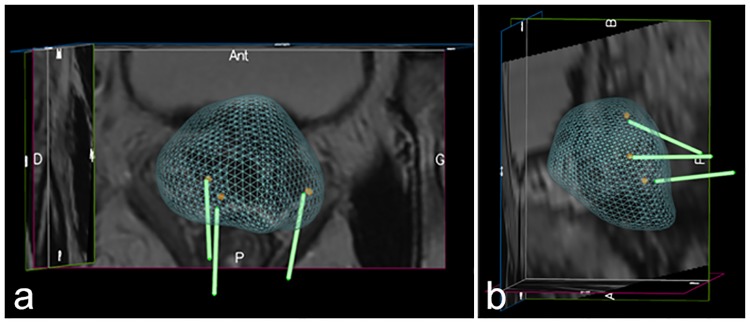
Three dimensional co-registration images showing the relative position of the virtual biopsies and the MR targets from an inferior (A) and lateral (B) perspective. The gap between the tip of the virtual biopsies and the center of the MR targets shows the error in the co-registration process.

Table 2 shows TRE_AP_, TRE_RL_, TRE_HF_, TRE_2D_ and TRE_3D_ for the three standardized locations of the markers and the four readers. When all markers were taken into account, median TRE_2D_ and median TRE_3D_ were respectively 3.1 mm (IQR, 1.9–3.7) and 3.8 mm (IQR, 2.8–5.8) for operator 1, 2.7 mm (IQR, 2.0–3.6) and 4.5 mm (IQR, 3.3–5.8) for operator 2, 2.5 mm (IQR, 1.6–4.5) and 4.2 mm (IQR, 2.9–5.6) for operator 3, and 3.6 mm (IQR, 2.4–4.5) and 5.6 mm (IQR, 3.6–7.1) for operator 4 (Fig 3).

**Table 2:** Overall target registration error.

		Apex					Midgland					Base				
		TRE_RL_	TRE_AP_	TRE_HF_	TRE_2D_	TRE_3D_	TRE_RL_	TRE_AP_	TRE_HF_	TRE_2D_	TRE_3D_	TRE_RL_	TRE_AP_	TRE_HF_	TRE_2D_	TRE_3D_
Op 1	Med (mm)	1.0	4.4	2.7	3.2	5.4	1.4	2.1	1.8	3.1	3.3	1.2	1.1	1.9	2.7	2.9
	IQR (mm)	0.5–1.8	2.9–6.7	0.7–4.3	2.2–3.6	3.6–6.9	0.8–2.5	0.8–3.8	0.5–3.0	1.9–3.7	2.9–5.3	0.8–1.9	0.7–3.0	0.6–2.5	1.9–3.3	2.2–4.4
Op 2	Med (mm)	1.7	5.1	1.4	3.0	5.7	1.6	2.0	1.7	2.6	3.8	1.2	1.7	1.9	2.9	4.0
	IQR (mm)	0.7–2.4	3.6–5.9	0.8–3.4	2.4–3.6	5.0–6.1	0.6–2.4	1.2–3.7	0.9–2.2	2.0–3.3	2.8–4.9	0.9–2.2	1.0–2.5	1.3–2.8	1.8–3.6	3.2–5.4
Op 3	Med (mm)	1.2	3.9	1.5	2.2	4.7	1.2	1.6	1.6	2.0	3.6	1.7	1.6	1.6	2.9	4.3
	IQR (mm)	0.2–2.0	3.7–4.4	0.4–3.0	1.1–4.5	3.8–5.9	0.5–1.8	0.7–3.5	0.8–3.4	1.6–4.6	2.3–5.3	1.0–2.8	0.4–3.0	0.5–2.8	2.2–4.3	2.8–5.0
Op 4	Med (mm)	2.79	5.08	1.43	3.3	6.5	1.37	2.79	2.97	3.6	4.9	1.56	1.30	1.84	3.5	4.4
	IQR (mm)	1.4–4.0	2.3–6.3	0.7–2.1	2.7–4.6	4.7–7.2	0.6–2.2	1.3–4.2	1.1–3.9	3.6–4.6	3.4–7.5	1.1–3.8	0.7–2.2	1.0–3.4	3.5–3.9	2.9–6.0

TRE: Target Registration Error; Op: Operator; Med: Median; IQR: Interquartile range.

TRE_3D_ was significantly different between operators (p = 0.013), although operators 1, 2 and 3 obtained close values of mean TRE_3D_. As compared to operator 3, the difference in mean TRE_3D_ for operators 1, 2 and 4 was +0.07 mm (95% confidence interval (95%CI): -0.93; +1.07), +0.21 mm (95% CI: -0.79; +1.21) and +1.47 mm (95% CI: +0.47; +2.47) respectively.

When the operator effect was taken into account, TRE_3D_ was significantly influenced by the location of the marker (p = 0.001), but not by the prostate volume (p = 0.25). Mean TRE_3D_ was smaller in the mid-gland and base than in the apex, with an average difference of -1.21 mm (95% CI: -2.03; -0.4) and -1.56 (95% CI: -2.44; -0.69) respectively.

When the effects of the operator, prostate volume and markers location were taken into consideration, TRE_3D_ was significantly different along the three directions of space (p<0.0001). TRE_AP_ and TRE_HF_ were larger than TRE_RL_, with an average difference of +1.29 mm (95% CI: +0.87; +1.71) and +0.52 mm (95% CI: +0.1; +0.95) respectively.

Median overall FLE_US_ and FLE_MR_ were respectively 0.6 mm (IQR, 0.4–1.0) and 0.9 mm (IQR, 0.9–1.2). They were respectively 0.6 mm (IQR, 0.4–1.5) and 0.9 mm (IQR, 0.7–1.2) for the apex markers, 0.5 mm (IQR, 0.4–0.9) and 1.0 mm (IQR, 0.5–1.2) for the mid-gland markers, and 0.7 mm (IQR, 0.4–1.0) and 0.8 mm (0.6–1.0) for the base markers.

## Discussion

Several difficulties may explain the lack of evaluation of the co-registration accuracy of commercially-available MR/US fusion systems in clinical routine. First, reliable landmarks are lacking. Anatomical landmarks, visible on both MR and US images, may be difficult to find or may be too large to serve as precise markers. Second, co-registration assessment needs that a common system of coordinates for co-registered US and MR volumes is saved and stored, which is not possible for all platforms. In this study, we took advantage of new fiducials that are easily visible on US and MR images. We also took advantage of the Urostation^™^ platform [11, 12, 27–30] that has three advantages: (i) it can save the coordinates of ROIs within a single volume for MR and US data, (ii) it performs elastic fusion based only on the US and MR prostate outlines, allowing the fiducials, that are not used for fusion, to be used as co-registration accuracy markers, and (iii) it allows retrospective fusion which made possible a multi-operator study.

Depending on the operator, median TRE_3D_ ranged from 3.8 to 5.6 mm, which is larger than the TREs published in preclinical studies for elastic fusion methods. This is not surprising since, as explained above, preclinical studies are likely to underestimate the co-registration errors made in daily routine.

The largest error was in the AP direction, i.e. along the probe axis. This may be due to the fact that the prostate is the most deformed in this direction by rectal movements or by the pressure applied by the probe. This error is less problematic for biopsy purposes since the AP direction is the direction of the needle track and since the biopsy needle samples 15–20 mm along its course. What matters most is the error in the plane orthogonal to the biopsy track (TRE_2D_), which ranged from 2.5 to 3.6 mm in our study. These error values are reasonable for clinical practice and remain substantially inferior to the radius (5 mm) of a 0.5-cc tumor, a volume usually considered the threshold for clinically significant prostate cancers [31].

TRE should always be interpreted with FLE that estimates the minimal error measurable given markers conspicuity and image resolution. We obtained inframillimeter median FLE values on the US and MR images. This good reproducibility reflects the good conspicuity of the markers and validates our method.

TRE_3D_ was significantly different among operators. Operator 4, who had the least experience in prostate contouring, obtained significantly larger median TRE_3D_ than the three others. This suggests that expertise remains important in MR/US fusion results.

The markers location also significantly influenced TRE_3D_. The worst results were obtained in the apex. This finding is not surprising since the contouring of the apex is subjective and difficult.

Our study has some limitations. First the number of patients is limited. Second, as specified above, the Urostation^™^ systems performs two types of fusion: an automatic elastic US/US fusion that co-registers the position of each biopsy cores within the panorama volume, and an elastic MR/US fusion the accuracy of which depends on the operator’s outlines of the prostate on MR and US images and that co-registers the position of MR targets in the panorama volume. We measured the global TRE, but our study design cannot define the relative contribution of both fusions in this global error. However, the US/US fusion provided by the Urostation system has been evaluated on 786 registrations in an earlier study [23] and showed a registration failure of 2%. Third, any migration of the fiducials between the US and MR image acquisitions may have induced an overestimation of TRE. To minimize this risk we tried to perform US and MRI the same day. This could be achieved in all patients but one. Furthermore, the displacement of intraprostatic fiducials has been shown to be less than one millimeter within the course of radiotherapy [25, 26, 32]. Fourth, we used standard 3-mm thick MR images for fusion. This is likely to have increased the observed registration error, as compared to the use of a 3D isotropic MR acquisition. However, in daily routine, many MR/US fusions are performed without dedicated isotropic acquisitions. Potential improvement induced by an isotropic MR acquisition on registration error remains to be evaluated. Fifth, although we wanted to evaluate the fusion accuracy in conditions as close as possible to routine practice, the use of the fiducials imposed to obtain the MRI after the fiducials placement, and, thus, to perform a retrospective fusion. Therefore, our study does not fully mimic the time constraints of daily practice, which may influence the fusion accuracy.

In conclusion, we evaluated the accuracy of the MR/US fusion using the Urostation^™^ platform, using standard 3-mm MR slices in prospectively-included unselected patients. We obtained a median overall registration error of 3.8–5.6 mm and a median error in the plane orthogonal to the virtual biopsy track of 2.5–3.6 mm. This seems reasonable for clinical practice. Experience significantly influenced fusion accuracy. The registration error remained significantly larger in the apex and it may be necessary to increase the number of targeted biopsies in this part of the gland, especially if the MR target is small.

## Supporting Information

S1 Table Patients’ characteristics and coordinates of individual MR targets and virtual biopsy tips and bases. (XLSX)
